# Supplementary material for: Mutation in ROBO3 Gene in Patients with Horizontal Gaze Palsy with Progressive Scoliosis Syndrome: A Systematic Review
Source: Int J Environ Res Public Health. 2020 Jun 22;17(12):4467. doi: 10.3390/ijerph17124467 (PMC7345006; doi:10.3390/ijerph17124467)
Supplement: Supplementary file 1 [file ijerph-17-04467-s001.pdf]

Table 1: Quality Assessment Tool for Case Series Studies

Author (year)	Q 1	Q 2	Q 3	Q 4	Q 5	Q 6	Q 7	Q 8	Q 9
Steffen et al.[18] (1998)	Yes	Yes	Yes	Yes	NA	Yes	Yes	NA	Yes
Jen et al.[8] (2002)	Yes	Yes	Yes	Yes	NA	Yes	NA	NA	Yes
Pieh et al.[12] (2002)	No	Yes	Yes	Yes	NA	Yes	NA	NA	Yes
Lo et al.[19] (2004)	Yes	Yes	Yes	Yes	NA	No	NA	NA	No
Rossi et al.[27] (2004)	Yes	Yes	NA	NA	NA	No	NA	NA	No
Incecik et al.[20] (2005)	Yes	Yes	Yes	Yes	NA	Yes	Yes	NA	Yes
Chan et al.[21] (2006)	Yes	Yes	Yes	Yes	Yes	Yes	Yes	NA	Yes
Dos Santos et al.[28] (2006)	Yes	Yes	NA	NA	NA	Yes	NA	NA	Yes
Haller et al.[29] (2008)	Yes	Yes	No	NA	NA	Yes	NR	NA	Yes
Abu-Amero et al.[43] (2009)	Yes	No	No	Yes	NA	Yes	Yes	NA	Yes
Amouri et al.[4] (2009)	Yes	No	No	Yes	NA	No	No	NA	Yes
Bomfim et al.[30] (2009)	Yes	Yes	NA	NA	NA	Yes	NR	NA	No
Avadhani et al.[31] (2010)	Yes	Yes	NA	NA	NA	Yes	NR	NA	Yes
Abu-Amero et al.[32] (2011)	Yes	Yes	No	Yes	NA	Yes	Yes	NA	Yes
Abu-Amero et al.[14] (2011)	Yes	Yes	NA	NA	NA	Yes	NR	NA	Yes
Jain et al.[23] (2011)	Yes	No	Yes	Yes	NA	Yes	NR	NA	Yes
Ng et al.[11] (2011)	Yes	Yes	NA	NA	NA	Yes	NR	NA	Yes
Volk et al.[33] (2011)	Yes	Yes	No	Yes	NA	Yes	Yes	NA	Yes
Bakbak and Kansu[24] (2012)	Yes	Yes	No	Yes	NA	Yes	Yes	NA	Yes
Kurian et al.[25] (2013)	Yes	Yes	No	Yes	NA	Yes	No	NA	Yes
Pina et al.[34] (2014)	Yes	Yes	NA	NA	Yes	No	Yes	NA	No
Bozdoğan et al.[35] (2017)	No	Yes	NA	NA	Yes	Yes	NR	NA	Yes
Mendes Marques et al.[26] (2017)	Yes	Yes	NA	Yes	NA	Yes	NR	NA	Yes
Lin et al.[36] (2018)	Yes	Yes	NA	NA	Yes	Yes	NR	NA	Yes
Rousan et al.[3] (2019)	Yes	Yes	No	Yes	NA	Yes	NR	NA	Yes

Question 1: Was the study question or objective clearly stated?; Question 2: Was the study population clearly and fully described, including a case definition?; Question 3: Were the cases consecutive?; Question 4: Were the subjects comparable?; Question 5: Was the intervention clearly described?; Question 6: Were the outcome measures clearly defined, valid, reliable, and implemented consistently across all study participants?; Question 7: Was the length of follow-up adequate?; Question 8: Were the statistical methods well-described?; Question 9: Were the results well-described?; Q: Question; CD: Cannot determine; NA: Not applicable; NR: Not reported.
